# Extracellular vesicles mediate biological information delivery: A double-edged sword in cardiac remodeling after myocardial infarction

**DOI:** 10.3389/fphar.2023.1067992

**Published:** 2023-02-22

**Authors:** Peipei Cheng, Xinting Wang, Qian Liu, Tao Yang, Huiyan Qu, Hua Zhou

**Affiliations:** ^1^ Institute of Cardiovascular Disease of Integrated Traditional Chinese and Western Medicine, Shuguang Hospital Affiliated to Shanghai University of Traditional Chinese Medicine, Shanghai, China; ^2^ Branch of National Clinical Research Center for Chinese Medicine Cardiology, Shuguang Hospital Affiliated to Shanghai University of Traditional Chinese Medicine, Shanghai, China; ^3^ Department of Cardiovascular Disease, Shuguang Hospital Affiliated to Shanghai University of Traditional Chinese Medicine, Shanghai, China

**Keywords:** myocardial infarction, cardiac remodeling, extracellular vesicles, cargo delivery, drug delivery

## Abstract

Acute myocardial infarction (AMI) is a severe ischemic disease with high morbidity and mortality worldwide. Maladaptive cardiac remodeling is a series of abnormalities in cardiac structure and function that occurs following myocardial infarction (MI). The pathophysiology of this process can be separated into two distinct phases: the initial inflammatory response, and the subsequent longer-term scar revision that includes the regression of inflammation, neovascularization, and fibrotic scar formation. Extracellular vesicles are nano-sized lipid bilayer vesicles released into the extracellular environment by eukaryotic cells, containing bioinformatic transmitters which are essential mediators of intercellular communication. EVs of different cellular origins play an essential role in cardiac remodeling after myocardial infarction. In this review, we first introduce the pathophysiology of post-infarction cardiac remodeling, as well as the biogenesis, classification, delivery, and functions of EVs. Then, we explore the dual role of these small molecule transmitters delivered by EVs in post-infarction cardiac remodeling, including the double-edged sword of pro-and anti-inflammation, and pro-and anti-fibrosis, which is significant for post-infarction cardiac repair. Finally, we discuss the pharmacological and engineered targeting of EVs for promoting heart repair after MI, thus revealing the potential value of targeted modulation of EVs and its use as a drug delivery vehicle in the therapeutic process of post-infarction cardiac remodeling.

## 1 Introduction

Among the leading causes of death and disability worldwide are acute myocardial infarction (AMI) and end-stage heart failure (HF), causing approximately 17 million deaths annually and accounting for 30% of all deaths globally (Ong et al., 2018; Roth et al., 2020). Coronary atherosclerosis underlies the development of myocardial infarction (MI), which contributes to vessel lumen narrowing and ultimately elicits myocardial necrosis due to hypoxia/ischemia. Currently, the primary goal of clinical treatment for such disease is to promptly restore flow in the occluded vessel and restore the ischemic myocardium, commonly performed by direct percutaneous coronary intervention. Intriguingly, however, such interventions can contribute to cardiomyocyte death and myocardial injury (Schirone et al., 2022). Consequently, novel therapies and interventions are urgently required to attenuate adverse ventricular remodeling and mitigate HF.

The heart undergoes a series of structural and functional alterations to adapt to cardiac injury induced by MI, which is known as cardiac remodeling (Zhao et al., 2020). Specifically, cardiac remodeling is a complex pathophysiological process, including inflammation, mitochondria dysfunction, oxidative stress, cardiac hypertrophy, and cardiac fibrosis. The entire process can be divided into two stages, the inflammatory phase and the fibrotic scar revision phase (Schirone et al., 2017; Liu H et al., 2020). The initial phase involves the exposure of intracellular contents into the extracellular environment due to cell necrosis, called damage-associated molecular patterns (DAMPs), triggering and exacerbating sterile inflammation. While the latter phase is attributed to the transdifferentiation of fibroblasts into myofibroblasts stimulated by multiple factors, including inflammatory factors, angiotensin II, and mechanical stress stimulation, which secrete collagen and facilitate scar repair in the infarcted region and maintain the structural integrity of the ventricle (Prabhu and Frangogiannis, 2016; Peet et al., 2020).

During cardiac remodeling, numerous immune cells recruitment to the infarct area, interact with cardiac fibroblasts (CFs) which crucially coordinates the balance between profibrotic and antifibrotic effects (Zaidi et al., 2021). In the initial inflammatory phase of AMI, the recruitment and activation of immune cells, such as macrophages, neutrophils, and monocytes, not only secrete inflammatory mediators to aggravate the inflammation but also shift towards an anti-inflammatory phenotype, which can prevent the release of their contents into the extracellular environment by removing necrotic cellular debris from the MI zone to attenuate inflammation (Ong et al., 2018; Andreadou et al., 2019; Doran et al., 2020). Besides this, the crosstalk between other diverse cells, including mesenchymal stem cells, endothelial cells (ECs), and cardiomyocytes is also essential for maintaining dynamic balance throughout the whole process. Interestingly, however, these cells secrete large numbers of EVs, which carry numerous bioactive substances, including miRNAs, lipids, and proteins. The bioactive substances not only have similar roles to parental cells but are also essential regulators of intercellular communication (Xiong et al., 2021). In general, there are three major subtypes of EVs, apoptotic bodies (ABs), microvesicles (MVs), and exosomes (Sanchez-Alonso et al., 2018), of which the most comprehensively explored are exosomes. In contrast, the least well-studied are ABs.

This review summarizes the dual roles of EVs derived from diverse cells in postinfarction cardiac remodeling, including pro-inflammatory and anti-inflammatory effects in the initial postinfarction inflammatory response and profibrotic and antifibrotic effects in the scar repair phase. Importantly, we discuss the role of crosstalk between these EVs in achieving a coordinated dynamic balance between the inflammatory response and the fibrotic process, which is significant for maladaptive cardiac remodeling after MI. In parallel, we also discuss therapeutic opportunities for targeting EVs that play a significant role in post-infarction cardiac remodeling, including pharmacological preconditioning and engineered modification. These elements will be beneficial in expanding therapeutic strategies for post-infarction cardiac remodeling.

## 2 Pathophysiological process of cardiac remodeling after myocardial infarction

AMI results from myocardial blood flow obstruction due to atherosclerotic plaque rupture or erosion (Ibanez et al., 2018). AMI is currently treated mainly by revascularization procedures such as thrombolysis and coronary intervention. However, recanalizing occluded vessels under long-term ischemic conditions causes additional myocardial damage, termed “I/R injury” (Yellon and Hausenloy, 2007). Such a process is accompanied by a blockage of blood flow, a sudden and dramatic interruption of oxygen and nutrient supply in the blood, a short cessation of mitochondrial oxidative phosphorylation (Frank et al., 2012), a dramatic decrease in available intracellular adenosine triphosphate (ATP), and a reduction in cardiomyocyte contractile function and cardiac stiffness and contracture minutes after the onset of ischemia (Jennings and Reimer, 1991). In the meantime, the myocardial ischemic injury may contribute to substantial cardiomyocyte death *via* different mechanisms. At the outset of reperfusion, cytosolic calcium overload and massive reactive oxygen species (ROS) generation rupture the mitochondrial membrane and release the contents, which in turn activate the caspase-mediated intrinsic pathway of apoptosis (Schirone et al., 2022). On the other hand, phosphorylation of receptor-interacting protein kinase 3 (RIPK3) is recruited and activated by the receptor-interacting protein kinase 1 (RIPK1), which in turn triggers necroptosis and exacerbate the pathological process after MI (Rodriguez et al., 2016).

The large volume of dead and injured cardiomyocyte contents (including heat shock proteins, organelle debris, and nuclear debris) enter the interstitial matrix, namely, DAMPs (Frangogiannis, 2012; Timmers et al., 2012). Simultaneously, innate immunity is triggered when pattern recognition receptors (PRRs) on leukocytes recognize DAMPs (Suetomi et al., 2019; Silvis et al., 2020). Immediately afterwards, the Toll-like receptor signaling pathway and other numerous inflammatory pathways are activated, thereby inducing proinflammatory cytokine expression, including interleukin 6 (IL-6), interleukin 1β (IL-1β), and tumor necrosis factor α (TNF-α), as well as the expression of chemokines, including monocyte chemotactic protein-1 (MCP-1/CCL2) (Frangogiannis, 2007). Additionally, neutrophils and inflammatory monocytes recruited by chemokine CCL2 activation are recruited to the infarcted heart (Dewald et al., 2005; Ley et al., 2007). On the other hand, when tissues are damaged, nuclear factor κB (NF-κB) is activated in ROS-dependent way and increases the expression of specific cellular genes, which can also eventually stimulate the growth of cells associated with inflammation and immune responses and exacerbate the inflammation even further (Kabe et al., 2005; Sun, 2009).

Short-term inflammatory responses contribute to debris removal, while long-term inflammation can facilitate extracellular matrix degradation and cell death, resulting in infarct size expansion. Consequently, timely resolution of the inflammation is essential for maintaining post-infarction remodeling. At the same time, apoptosis and clearance of neutrophils in the infarcted area is a hallmark that inflammation is beginning to subside (Frangogiannis, 2012; Westman et al., 2016; Ong et al., 2018). As leukocyte infiltration removes dead cells and matrix debris from the infarct and represses inflammatory mediator release, the anti-inflammatory monocyte subsets become dominant (Frangogiannis, 2008; Prabhu and Frangogiannis, 2016). Furthermore, suppressive subsets of other immune cells like lymphocytes, monocytes, and anti-inflammatory macrophages can suppress inflammation in the infarcted heart. Macrophages, in particular, play a crucial role in this process by shifting from a pro-inflammatory to an anti-inflammatory phenotype (Yan et al., 2013). In this way, the post-infarction pathological process moves from the pro-inflammatory response phase to the anti-inflammatory response phase, collectively referred to as the inflammatory response phase (within 3–7 days after MI), and eventually to the scar repair phase (7 days after MI) (Liu S et al., 2020).

In the cardiac tissue remodeling phase, fibroblast proliferation, extracellular matrix secretion, and eventually fibrotic scar maturation (Kologrivova et al., 2021). During the scar proliferation phase, subpopulations of monocytes and macrophages activate and recruit mesenchymal repair cells and primarily fibroblast-like cells by secreting growth factors (Frangogiannis, 2014). There is evidence that CFs originate from circulating progenitor stem cells (PSCs) recruited into the ventricular myocardium after birth and distribute throughout the normal myocardium as strands and sheets between myocardial muscle fibers and are critical effector cells in the scar repair process (Visconti and Markwald, 2006). In the normal physiological state, CFs account for 60%–70% of total cardiac cell numbers (Porter and Turner, 2009). They secrete collagen, which provides structural scaffolding for cardiomyocytes, coordinates myocardial motion, and mediates the onset of electrical activity (Bretherton et al., 2020). Under pathological conditions, fibroblasts transdifferentiate into myofibroblasts (MFBs) in response to various stimuli, including renin-angiotensin system activation, mechanochemical signaling, and release of transforming growth factor β (TGF-β), inflammatory factors (Frangogiannis, 2015; Han et al., 2022).

MFBs possess characteristics of both fibroblasts and smooth muscle cells, and secrete collagen to promote myocardial fibrosis. Meanwhile, these cells are analogous to smooth muscle cell contraction (as present in the vascular system), which can be modulated by circulating factors and neurohormones, including angiotensin II (van den Borne et al., 2010). Apoptosis of most repair cells indicates the end of the proliferative phase, and as the development of MI pathophysiology, the scar matures *via* a steady increase in collagen cross-linking (Frangogiannis, 2012). However, persistent MFBs differentiation and activation, as well as extracellular matrix (ECM) deposition, contribute to a decreased compliance of the myocardium (Umbarkar et al., 2021). On the other hand, progressive decline in the delivery of oxygen and nutrients induces atrophy and cell death in the cardiomyocytes, contributing to progressive acute left ventricular dilatation and, ultimately, dysfunction and heart failure (Gibb et al., 2020). Thus, controlling the fibrosis process and achieving a dynamic balance between pro-and anti-fibrosis are of great significance for post-infarction repair. Given the complexity of post-infarction cardiac remodeling, this review focuses on two significant aspects: inflammatory response and fibrotic scar repair.

## 3 Biogenesis, sorting, delivery, and outcome of extracellular vesicles

EVs are nanoscale lipid particles generated by the endosomal system of eukaryotes and secreted into the extracellular environment (Borges et al., 2013; van der Pol et al., 2014; Yanez-Mo et al., 2015; Zaborowski et al., 2015). Currently, there is still no exact criteria on the nomenclature of EVs. Various names are used, such as exosomes, microparticles, MVs, ABs. Remarkably, the nomenclature of “extracellular vesicles” proposed by Chistiakov mainly used in this review (Chistiakov et al., 2015), which means that EVs are typically categorized subsets by their size, formation, marker proteins, release mechanisms, and lipid composition, including three types of plasma membrane exfoliated vesicles: exosomes (30–150 nm), MVs (1,000–1,000 nm), and ABs (500–2,000 nm) (Liu H et al., 2021). Exosomes are phospholipid bilayers membrane-enclosed vesicles (Pegtel and Gould, 2019), which were initially discovered in sheep reticulocytes in 1983 and then were named “exosomes” by Johnstone in 1987 (Pan and Johnstone, 1983; Johnstone et al., 1987). Several studies have indicated that the inward budding of the early endosomal membrane forms early endosomes and then matures into late endosomes which are often packed with small intraluminal vesicles (ILVs) and evolve into multivesicular bodies (MVBs) (Ohayon et al., 2021). After a series of intracellular transport, the degradation of vesicles occurs when some MVBs fuse with lysosomes. While other MVBs release ILVs such as exosomes when fusing with the plasma membrane (Kowal et al., 2014; Villarroya-Beltri et al., 2016). These exosomes can be detected in diverse bodily fluids, such as breast milk, saliva, blood, urine (Pisitkun et al., 2004; Caby et al., 2005; Admyre et al., 2007; Palanisamy et al., 2010). Simultaneously, numerous studies suggest that a broad range of cell types, including cardiomyocytes, immune cells, progenitor cells, fibroblasts, and stem cells can generate exosomes (Zitvogel et al., 1998; Doyle and Wang, 2019).

MVs were first described by Peter Wolf in 1967 and are generated by the outward budding of plasma membrane (Wolf, 1967), and they are also the result of dynamic interaction between redistribution of phosphatidylserine (PS) and the remodeling of cytoskeletal proteins (Zwaal and Schroit, 1997; Leventis and Grinstein, 2010); proteins and phospholipids are distributed very heterogeneously and form microdomains within the membrane. Aminophospholipid translocases tightly control this asymmetric distribution of PS on plasma membranes. Moreover, flippase is a translocase that translocates PS from the outer leaflet to the inner leaflet of the membrane, while floppase transfers phospholipids from the inner to the outer leaflet of the plasma membrane (Zwaal and Schroit, 1997; Hugel et al., 2005). Immediately afterwards, actin-myosin interactions promote cytoskeleton contraction, aid cell membranes’ budding, and ultimately form MVs (McConnell et al., 2009; Muralidharan-Chari et al., 2009). The number of these generated MVs relies on the physiological state and microenvironment of the donor cells while consumed MVs depends on the physiological state and microenvironment of the recipient cells (Zaborowski et al., 2015).

**FIGURE 1:**
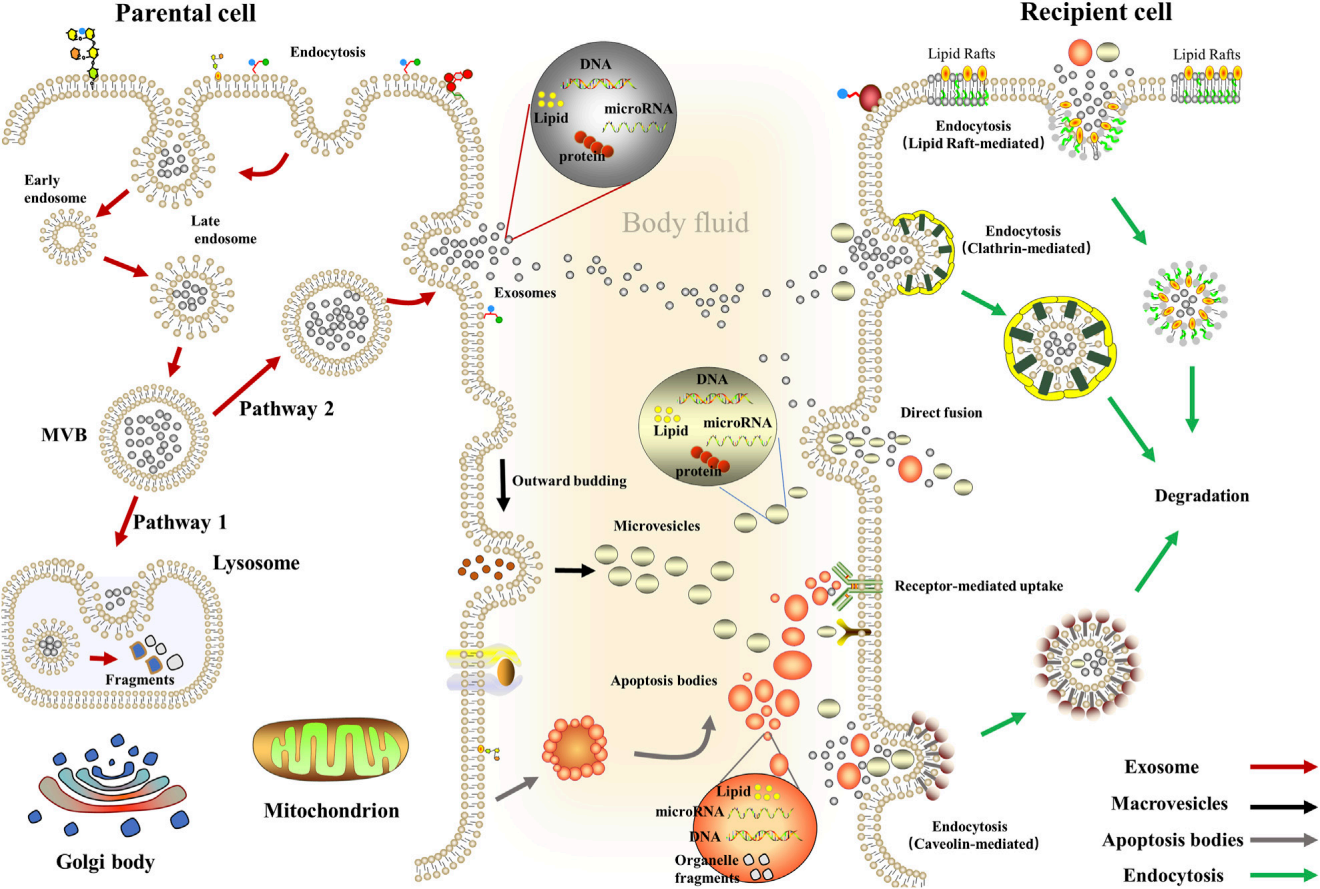
Biogenesis and delivery of extracellular vesicles. Exosomes: Inward budding of the early endosomal membrane forms early endosomes and then matures into late endosomes and evolves into MVBs. Then, some MVBs fuse with lysosomes while other MVBs fuses with the plasma membrane to release exosomes (van Niel et al., 2018); Macrovesicles: Actin-myosin interactions promote cytoskeleton contraction, aid cell membranes’ budding, and ultimately form macrovesicles (McConnell et al., 2009); Apoptosis Bodies: The cytosolic membrane is wrinkled and invaginated, dividing and wrapping the cytoplasm, containing DNA material and organelles, forming Apoptosis Bodies (Liu J et al., 2018).

These EVs can enter the recipient cells *via* fusing with cellular membranes; on the other hand, it also enters the recipient cells *via* different endocytic pathways, including phagocytosis clathrin-mediated endocytosis, caveolae, or lipid raft-mediated endocytosis (Feng et al., 2010; Tian et al., 2014; Costa Verdera et al., 2017; Hou and Chen, 2021). Moreover, classical intercellular adhesion molecules also mediate the binding of EVs to the target cells (Zhao et al., 2015); specifically, on the one hand, TIM family members (TIM1, TIM4) PS transmembrane receptors are used by target cells for exosomes entry (Miyanishi et al., 2007), on the other hand, exosomes derived from B cells containing a 2,3-linked dialkylated glycoprotein can be captured by lymph node and spleen CD169^+^ macrophages (Saunderson et al., 2014). At the same time, macrophages uptake exosome *via* C-type lectins expressed in macrophages and galectin-5 exposed to exosome (Barres et al., 2010). Meanwhile, multiple mechanisms have been demonstrated to be associated with regulating the biogenesis of EVs, especially the endosomal sorting complex required for transport (ESCRT) or non-ESCRT mechanisms (Assil et al., 2015). ESCRT consists of four core subunits (ESCRT-0; ESCRT-I; ESCRT-II; ESCRT-III), where ESCRT-0 mediates substrate recognition and sorting, ESCRT-I and ESCRT-II are critical for mediating endosomal membrane inward budding, and ESCRT-III is responsible for shearing the neck of the budding body, thereby orchestrating the formation of an inward budding vesicle of MVBs (Hurley and Hanson, 2010; Hanson and Cashikar, 2012; Tarasov et al., 2021). Considering the biological complexity of EVs and is susceptible to the environment, there are yet no established criteria to distinguish different types of EVs, which limits the development of related studies (Amosse et al., 2017) (Figure 1).

## 4 Composition and physiological function of extracellular vesicles

Initially, EVs were considered waste products released by cells. Later studies, however, demonstrated that EVs mediate the transmission of intercellular molecular biological information and play a crucial role in intercellular communication (Hessvik and Llorente, 2018; Harmati et al., 2021). Besides, EVs can transport bioactive cargoes, including RNA, DNA, proteins, and lipids (Chen F et al., 2020). Indeed, it both retains many of the biological properties of their parental cells and regulates recipient cells’ physiological or pathological processes (Xing et al., 2021). Of the three types of EVs, exosomes are derived from MVBs, which budding inward from a raft-like domain and, therefore, have a slightly different lipid content than the cell membrane. Exosomes are rich in cholesterol, sphingomyelin, glycerophosphocholine, and phosphatidylcholine (Laulagnier et al., 2004; Raposo and Stoorvogel, 2013; Janas et al., 2015), and membrane surface markers such as integrins, Flotillin 1, CD63, CD81, Alix, TSG101, and cell adhesion molecules (CAMs) (Lotvall et al., 2014). Due to its small size, protective lipid bilayer, and surface receptors, exosome can mediate long-distance, inter-tissue systemic crosstalk (Mathieu et al., 2019; Pelissier Vatter et al., 2021).

The lipid composition and markers of MVs depend mainly on the composition of their cell membrane and also show particular antigens concerning the membrane composition of the original cell. For example, T cell MVs carry CD4 or T cell receptors (Choudhuri et al., 2014). Monocyte-derived MVs show CD14, while CD31, CD34, CD51, CD62E, CD144 and CD146 expressed on endothelium-derived MVs (Koga et al., 2005). Microvesicular cholesterol and hyperphosphatidylserine expose a lipid composition different from that of exosomes. Addition, the shape and density of MVs are irregular as well as the surface does not have any tetrapeptide (Muralidharan-Chari et al., 2010; Choudhuri et al., 2014). These vesicles consist of a lipid bilayer and have mRNAs, miRNAs, biologically active lipids, metabolites, and protein in which surface phospholipid bilayer (Ratajczak and Ratajczak, 2020), it also expresses the number of transmembrane proteins, such as integrins and selectins as in the parental cell membrane (Bobrie et al., 2011; Raposo and Stoorvogel, 2013). However, MVs contain a large number of cytoplasmic and plasma membrane proteins, such as cytoskeletal proteins, heat shock proteins, and protein tetrapeptides that accumulate on the surface of the plasma membrane, which is 100-fold more concentrated in MVs than in cell lysates (Escola et al., 1998; Zoller, 2009; Di Vizio et al., 2012; Morello et al., 2013), and contribute significantly to intercellular communication (Cocucci et al., 2009). On the other hand, the function of MVs depends on the cell types from which they originate (Muralidharan-Chari et al., 2010). For example, immune cell-derived MVs are involved in inflammatory responses, while endothelial cell-derived MVs are related to blood vessels (Morel et al., 2004). In addition to this, the procoagulant properties of MVs were reported for the first time in 1946 by Chargaff and West and later confirmed by Wolf in 1967, who described them as “platelet dust” (Wolf, 1967; Morel et al., 2004).

In recent years, there have been concerns regarding the relationship between EVs and diseases, with numerous studies focusing on cancer (Prieto-Vila et al., 2021), Parkinson’s (D'Anca et al., 2019), infectious diseases (Rodrigues et al., 2018), kidney diseases (Thongboonkerd, 2019), and cardiovascular diseases, resulting in substantial influential outcomes. A great deal of research in cardiovascular disease suggests that EVs play an essential role in cardiac pathophysiology (Njock et al., 2015), and EVs of diverse origins are necessary for maintaining cardiac function and homeostasis *via* influencing and interacting with each other (Yu and Wang, 2019). These subgroups exhibit a wide range of functions, making them an important source of potential biomarkers for early diagnosis, therapeutic drug delivery systems, or vaccine production systems (Bordanaba-Florit et al., 2021).

## 5 Mechanism of extracellular vesicles involved in cardiac remodeling after myocardial infarction

Numerous studies have shown that EVs have similar biological properties to parental cells. Interestingly, however, multiple cells produce EVs after MI, including cardiomyocytes, immune cells, ECs, progenitor cells, fibroblasts, and stem cells (Fujita et al., 2019; Berezin and Berezin, 2020). The presence of pro-inflammatory M1 macrophages (M1-MØ) and reparative M2 macrophages (M2- MØ) as well as N1 (Ly6G^+^CD206^-^) and N2 (Ly6G^+^CD206^+^) neutrophil phenotypes in the infarcted heart mean immune cells exhibit functions and phenotypic heterogeneity (Ma et al., 2016; Peet et al., 2020). N1 neutrophils are pro-inflammatory, with high expression of pro-inflammatory markers, such as TNF-α, IL-1b, macrophage inflammatory protein 1α (CCL3), and interleukin 12a (IL-12a), while anti-inflammatory Cd206 and interleukin 10 (IL-10) are highly expressed in N2 neutrophils. Similarly, monocytes differentiate into pro-inflammatory and anti-inflammatory. EVs of different origins and subtypes also have complex and diverse roles similar to their parental cells and play a dual role in post-infarction cardiac remodeling. These EVs maintain a dynamic balance between pro-and anti-inflammatory, pro-and anti-fibrotic. Thus, it is significant to investigate the involvement of EVs in post-infarction cardiac remodeling, providing new avenues to open up targeted therapeutic strategies.

### 5.1 Mechanism of extracellular vesicles associated with the initial inflammatory response following myocardial infarction

#### 5.1.1 Mechanism of extracellular vesicles associated with the pro-inflammatory response after myocardial infarction

After MI, the I/R injury contributes to the increased release of various EVs from the heart, which on the one hand, increases the expression of pro-inflammatory cytokines by promoting M1-type polarization of macrophages, on the other hand, these EVs generated by I/R injury stimulate the inflammatory response *via* transferring miR-155-5p to macrophages activating the JAK2/STAT1 pathway (Ge et al., 2021). Furthermore, a large number of monocytes infiltrate the infarct zone and differentiate into pro-inflammatory macrophages, which generated exosomes containing pro-inflammatory miRNAs. When transported into CFs, these exosomes promote significant expression of inflammatory cytokines, including TNF-α, CCL2, IL-1β, and IL-6 in CFs, thereby exacerbating the inflammatory response in the infarct area (Wang et al., 2017). On the other hand, M1 macrophage-secreted exosomes can also transfer specific miR-155 to ECs, preventing migration and proliferation, affecting angiogenesis and repair, which in turn exert a pro-inflammatory effect (Liu Y et al., 2020).

Beyond that point, as one of the most critical members of the immune cell family, neutrophils play an essential role in this phase. It is well known that neutrophils are professional phagocytes that help to clear necrotic cells. However, recent studies have shown that excessive neutrophil accumulation or delayed clearance of neutrophils is detrimental and can act by secreting EVs (van der Pol et al., 2016). Depending on the production mechanism, neutrophil-derived EVs can be classified into two subtypes: neutrophil-derived MVs (NDMVs) and neutrophil-derived trails (NDTRs). Pro-inflammatory miRNAs are highly expressed in the NDTRs, which are found in tissues where neutrophils migrate while inducing pro-inflammatory macrophage polarization and exacerbating the inflammatory response and cardiac injury (Ma, 2021; Youn et al., 2021). On the other hand, NDMVs can evoke an acute inflammatory response by stimulating ECs to produce inflammatory mediators, including MCP-1 and IL-6 and tissue factor as well as increase microvascular endothelial permeability (Mesri and Altieri, 1999; Ajikumar et al., 2019). Numerous factors influence the increase of neutrophils. Generally, myocardial injury mobilizes neutrophils rapidly from the spleen to the peripheral blood, where they engage in transcriptional activation prior to reaching the damaged area. AMI triggers the transient release of cardiomyocyte-and endothelium-derived EVs. Ly6C + monocytes infiltrating the infarcted heart are stimulated to release chemokines and inflammatory cytokines by these vesicles, exacerbating the local inflammatory response (Loyer et al., 2018). Further studies demonstrated that endothelial cell-derived EVs mediated neutrophil mobilization from the spleen *via* EC-EV-VCAM-1 after AMI and induced transcriptional activation of blood neutrophils to facilitate miRNA-126-mRNA targeting (Akbar et al., 2022). At the same time, ABs are produced when a substantial number of cells appear apoptotic in infarct zones. However, these ABs do not contribute to the release of intracellular contents and trigger an inflammatory response due to their intact membrane structure, while a large number of necrotic cell contents are secreted into the extracellular area to trigger a dangerous related pattern and trigger a severe inflammatory reaction (Anversa et al., 1998).

Besides this, the current clinical treatment of MI is mainly through percutaneous coronary intervention (PCI). Available studies demonstrate that post-PCI vascular inflammation involves complex interactions between multiple cell types, these cells release proinflammatory cytokines while recruiting monocytes and neutrophils for transendothelial migration to surrounding tissues (Tucker et al., 2021), and EVs, as an important medium of intercellular communication, may play an important role in this process (S et al., 2013). On the other hand, Neutrophils mediate platelet aggregation and thrombus formation, while activating endothelial cells, macrophages and T lymphocytes to release inflammatory cytokines and promote an inflammatory cascade response (Vaidya et al., 2021). However, whether these immune cells release EVs in the process and whether these EVs play a role remains to be explored. Given the current context, future research directions regarding EVs in the regulation of microvascular inflammation after PCI may focus on several directions. Firstly, EVs can be used as potential biomarkers to assess the degree of vascular inflammation; secondly, they may be critical mediators of intercellular communication, broadening the understanding of pathological mechanisms associated with the microvascular inflammation after PCI; thirdly, EVs can be used as drug carriers or targets to alleviate microvascular inflammation after PCI.

#### 5.1.2 Mechanism of extracellular vesicles associated with the anti-inflammatory response following myocardial infarction

Macrophages are functionally and subtypically polarized into the classically activated M1 phenotype and the alternatively activated M2 phenotype. Despite its simplicity, this classification is still more accepted at this stage. M2 phenotype macrophages can mediate inflammation resolution by secreting anti-inflammatory mediators during days 3–5 following MI. Furthermore, it has been shown that the co-culture of the MSCs and macrophages can contribute to sustained high expression of CD206 and IL-10 and low expression of interleukin 12 (IL-12) and TNF-α on the surface of macrophages, resulting in a shift of macrophages towards the M2 phenotype (Chiossone et al., 2016). It has also been shown that mesenchymal stroma-derived exosomes promote macrophage polarization toward the M2 phenotype, which may attenuate the inflammatory cascade response and enhance subsequent repair activity, thereby limiting the infarct size. The mechanism is primarily associated with a large number of miR-182 transported in these exosomes, which regulate the phenotypic transition of macrophages *via* the TLR4/NF-κB pathway (Zhao J. et al., 2019). These studies suggest that exosomes of different cellular origins that interact with macrophages may be one of the mechanisms by which the myocardium is protected.

NDMVs are involved in the anti-inflammatory response as well as the pro-inflammatory response. These EVs have been demonstrated to have a significant anti-inflammatory effect on interacting cells, mainly by reducing the production of activated cytokines, including IL-1β, TNF-α, IL-6, IL-10, and IL-12 (Byrne and Reen, 2002; Eken et al., 2008; Ren et al., 2008; Eken et al., 2010; Alvarez-Jimenez et al., 2018). Apart from this, neutrophils exert their anti-inflammatory action by secreting EVs, such as NDMVs containing anti-inflammatory miRNAs in foci of inflammation (Youn et al., 2021). At the same time, the administration of neutrophil-derived EVs carrying membrane-linked protein A1 (AnxA1) inhibited inflammation. Overexpression of AnxA1 activated the STAT3 signaling pathway to inhibit neutrophil infiltration during myocardial I/R injury (Dalli et al., 2008; Zhao C. et al., 2019). On the other hand, it has been shown that leukocyte-derived MVs (LMVs) impede the leukocyte inflammatory responses to lipopolysaccharide and stimulate anti-inflammatory cytokine transforming growth factor β1 (TGF-β1) secretion (Gasser and Schifferli, 2004). Specifically, membrane-linked protein 1 (an anti-inflammatory protein) expressed on the surface of these particles exerts this effect (Dalli et al., 2008). *In vitro*, the particles are taken up by B cells and monocytes, thereby modulating their activation to an anti-inflammatory phenotype. Such phenomenon mediates the immune cells’ anti-inflammatory response in the early phase of acute infarction (Koppler et al., 2006).

Regulatory T (Treg) cells from the thymus or peripheral lymphoid organs are a subtype of T-cells (Richards et al., 2015). Inflammatory cell infiltration and macrophage polarization can be improved through paracrine action and direct contact. It inhibits cardiomyocytic apoptosis and is considered a potential target for reducing the inflammatory response to myocardial I/R injury. Recent studies have indicated that miRNA-181a is associated with immune regulation in cardiovascular disease. Furthermore, Wei et al. showed that miRNA-181a overexpression significantly suppressed inflammatory responses and increased Treg cell ratios by targeting the critical inflammatory transcription factor c-Fos. Additionally, miRNA-181a also attenuated the activation of dendritic cells (DCs) stimulated by oxidized low-density lipoprotein (ox-LDL). It is evident that miRNA-181a delivered by MSC-derived exosomes (MSC-Exos) combines the immunosuppressive effect of miRNA-181a with the cellular targeting ability of MSC-Exos and ultimately exerts a more substantial therapeutic effect on myocardial I/R injury (Wei et al., 2019).

Bone marrow mesenchymal stem cell (BMMSC) transplantation is considered potential therapeutic approach for MI; however, the body’s immune response can contribute to significant early death of transplant cells (Karantalis et al., 2014). Increasing evidence points specifically to the BMMSC-derived exosomes have immunomodulatory, anti-inflammatory effects and can be used as an alternative to stem cell transplantation (Moghaddam et al., 2019). The NF-κB pathway acts as a coordinator in inflammation and inhibiting the NF-κB pathway prevents ventricular remodeling and cardiac rupture in mice with acute myocardial infarction (Pramanik et al., 2018). EVs of BMMSCs can regulate the inflammatory microenvironment *via* regulating the BCL6/MD2/NF-κB signaling pathway, thereby delivering miR-302d-3p and attenuating the inflammatory response during cardiac remodeling after AMI (Liu et al., 2022). At the same time, it has also been shown that FNDC5 pretreated BMMSCs can secrete more exosomes that reduce the secretion of pro-inflammatory cytokines by macrophages while increasing the secretion of anti-inflammatory cytokines. MSCs-Exo and FNDC5-MSCs-Exo lessen the infiltration of inflammatory cells in ischemic heart tissue and improve the secretion of pro-inflammatory cytokines, thereby improving the inflammatory response in the heart early after myocardial infarction (Ning et al., 2021).

In addition, adipose mesenchymal stem cell-derived exosomes (ADMSC-Exos) exert anti-inflammatory effects by promoting macrophage M2 polarization and activating the S1P/SK1/S1PR1 signaling pathway to improve the inflammatory response in MI (Deng et al., 2019). Adipose stromal cells (ADSCs) can reduce MI severity (Kim et al., 2017), and ADSCs derived exosomes overexpression miR-126 have previously been shown to reduce inflammatory cytokines secreted by damaged cardiomyocytes (Luo et al., 2017). Furthermore, exosomes from miR-93-5p overexpressing ADSCs further targete TLR4 to downregulate the expression of inflammatory cytokines such as IL-6, IL-1β, and TNF-α in hypoxia-treated cardiomyocytes, thereby reducing autophagy-related proteins (LC3II and Atg7) expression and inflammation-related proteins (TLR4 and NF-κB p65) in cardiac tissue and ultimately alleviated inflammatory response (Liu D et al., 2018; Pan W et al., 2019).

At the same time, further work in this field of research contributes to paramount observations or considerations to pre-clinical research using tissue engineering or matrices carrying multifunctional MSC-EVs as an alternative therapeutic approach with associated anti-inflammatory or fibrotic benefits. EVs from porcine cardiac adipose tissue-derived MSC (cATMSC) combined with biocompatible cardiac scaffolds are capable of effective delivery in post-infarct myocardial tissue, decreasing expression of inflammatory TNF-α, increasing expression of IL-1ra, blocking IL-1α/β inflammatory effects and inducing anti-inflammatory IL-10 expression, which in turn attenuates the local inflammatory response in the myocardium. Meanwhile, the reduction of inflammatory mediators modulates the expression and activity of TGF-β, metallopeptidases, and other pro-fibrotic mediators, thereby reducing collagen synthesis and deposition and myocardial vascularization and fibrosis (Monguio-Tortajada et al., 2021; Monguio-Tortajada et al., 2022). Yet producing effective formulations of EVs and meeting the requirements for standardized clinical-grade biomanufacturing and regulatory, large-scale, GMP-compliant issues for EV production and clinical application remain a huge challenge (Courageux et al., 2022; Soler-Botija et al., 2022).

### 5.2 Mechanism of extracellular vesicles involved in scar repair after myocardial infarction

#### 5.2.1 Mechanism of extracellular vesicles involved in promoting scar repair

CFs are the primary effector cells in the scar repair phase of myocardial fibrosis, which transdifferentiate to MFBs in response to different stimuli, secrete collagen, and are the primary source of ECM. The scar repair phase involves several processes such as pro-angiogenesis and pro-differentiation of CFs into MFBs. CircUbe3a from M2 macrophage-derived exosomes promotes the proliferation or migration of CFs, and transdifferentiation *via* the miR-138-5p/RhoC axis, thereby mediating post-infarction myocardial fibrotic repair (Wang Y. et al., 2021). On the other hand, EVs generated from cardiosphere-derived cells (CDC-EVs) can polarize M1-MØ to a proangiogenic phenotype dependent on upward regulation of arginase to contribute to scar repair (Mentkowski et al., 2020). Moreover, several studies suggested that the expansion and activation of CD4^+^ T-cells in the heart associated with post-infarction remodeling. Exosomes generated from activated CD4^+^ T-cells carrying miR-142-3p, which mediates local MFBs activation through the miR-142-3p-WNT signaling cascade, secreting TGF-β, and regulating the APC-GSK-β-linked protein signaling cascade to confer a profibrotic effect. Aggravates post-ischemic myocardial fibrosis (Cai et al., 2020). Though EVs affect the post-infarction microenvironment by transporting miRNAs, cardiovascular risk factors, including sex, age, genetics, smoking, overweight, and obesity, affect apoptosis and activation of the mother cells of EVs, which in turn affect the physicochemical properties of these vesicles. Thus, most miRNAs are transported by cell-free and EV-free.

Cardiomyocyte-derived exosomes facilitate the formation of myocardial fibrosis *via* cardiomyocyte-fibroblast interactions. Previous study demonstrated that cardiomyocyte-derived exosomes enriched with miR-208a can promote the proliferation and differentiation of fibroblast toward MFBs (Yang et al., 2018). The molecular analysis then showed that miR-208a blocked the expression of Dyrk (bispecific tyrosine phosphorylation kinase), thereby upregulating fibrotic gene expression *via* the nuclear translocation of NFAT. Additionally, miR-217 was enriched in exosomes derived from cardiomyocyte and enhanced fibroblast activity by targeting the PTEN signaling cascade under pressure overload (Nie et al., 2018). At the same time, WNT3a in exosomes containing WNT protein can effectively trigger WNT/β-catenin signaling. In the presence of TGF-β, WNT3a can effectively enhance the pro-fibrotic response of human CFs (Dzialo et al., 2019). In addition, human umbilical cord mesenchymal stem cell-derived exosomes (hUCMSC-Exos) can promote CFs into FMBs and secrete collagen into inflammatory environment, thereby promoting cardiac fibrotic scar repair (Shi et al., 2019).

During the fibrotic repair phase of post-myocardial infarction, the precursors of ECs such as late endothelial progenitor cells (EPCs) can repair the heart through paracrine mechanisms. Several studies have demonstrated that human endothelial histocyte-derived exosomes enhance the proliferation and angiogenesis of CFs *in vitro* (Ke et al., 2017). In addition, cardiac mesenchymal stem cells (C-MSCs), a novel subpopulation of MSCs derived from cardiac tissue, have been shown to play an irreplaceable role in cardiac regeneration. Notch signaling is thought to contribute to cardiac repair after myocardial injury. EVs derived from Notch1 overexpressed C-MSCs are essential in promoting angiogenesis and cardiac fibroblast proliferation, preventing cell death, and facilitating myocardial fibrosis repair (Xuan et al., 2020).

Furthermore, in a MI model, it has been demonstrated that ABs released by transplanted MSCs can enhance angiogenesis and improve recovery of cardiac function by regulating macroautophagy/autophagy in recipient ECs. BMMSCs undergo extensive apoptosis immediately after transplantation and release ABs phagocytosed by recipient ECs. Then, these ABs activate lysosomal function and promote the expression of TFEB (transcription factor EB), the master gene for lysosomal biogenesis and autophagy in ECs. Notably, TFEB is involved in the expression of autophagy-related genes in ECs and boosts angiogenesis and recovery of cardiac function after MI (Liu H et al., 2020). It has been shown that myocardial endocytic target and silence CDIP1(cell death involved p53 target 1) gene *via* exosomal miRNA-21-5p, thereby downregulating activated caspase-3, which in turn inhibits apoptosis of ECs under ischemic and hypoxic conditions and facilitates post-MI angiogenesis and regeneration, which in turn involved in post-infarction scar repair (Liao et al., 2021).

#### 5.2.2 Mechanism of extracellular vesicles involved in the anti-scar repair

Scar repair is essential for maintaining cardiac structure in the early stage of MI, which involves angiogenesis and MFBs activation. Fibrotic scar repair is a double-edged sword, inadequate repair can contribute to cardiac rupture, and excessive and sustained fibrotic repair can lead to ventricular systolic-diastolic dysfunction and ultimately left heart failure (Ma et al., 2013; Ma et al., 2017). Harmonizing the dynamic balance of pro-and anti-fibrosis is crucial for post-infarction cardiac remodeling.

BMMSC-derived M1 macrophage exosomes inhibit angiogenesis and myocardial regeneration following MI by activating the MALAT1/miR-25-3p/CDC42 signaling pathway as well as MEK/ERK axis, thereby impeding scar repair (Chen et al., 2021). These EV-loaded miRNAs are significant for the pathological process of fibrosis in addition to their involvement in the early anti-inflammatory response to cardiac remodeling. Previous studies have indicated that MSCs derived EVs containing miR-212-5p may ameliorate myocardial fibrosis after MI by inhibiting the NLRC5/VEGF/TGF-β1/SMAD signaling pathway, as evidenced by reducing the expression of TGF-β1, collagen type I (Col I), and α-smooth muscle actin (α-SMA) (Wu et al., 2022). At the same time, MSC-derived extracellular vesicles (MSC-EVs) were also able to inhibit hypoxia-mediated cardiac fibroblast activation, in turn, exerting antifibrotic effects (Chen et al., 2014), secondly, these vesicles could be internalized by cardiac stem cells. Treatment of CSCs with MSC-Exo in an AMI model enhanced cell proliferation implantation and capillary density and reduced fibrotic area (Zhang et al., 2016). On the other hand, miR-199a-3p in exosomes released by BMMSCs targeted to the region of cardiac injury inhibited mTOR activation in myocardial tissue and subsequently activated induction of autophagy. Excessive autophagy eliminated the fibrotic fraction in the heart and attenuated tissue damage (Fan C et al., 2022). Concurrently, these exosomes can promote premature senescence of MFBs *in vitro* and enhance microvascular regeneration under stress, and ultimately improve cardiac function *via* modulating platelet-derived growth factor receptor-β (PDGFR-β) (Chen J et al., 2020; Wang et al., 2021a). Adipose-derived mesenchymal stem cells secreted miR-671-containing exosomes that attenuated myocardial fibrosis by inhibiting the TGFBR2/Smad2 signaling pathway (Wang et al., 2021b).

Cardiovascular progenitor cells (CVPCs) derived from human pluripotent stem cells are a promising source of myocardial repair with EVs mediating intercellular communication. Intramyocardial injection of HCVPC EVs into AMI mice on day 28 after MI significantly improved cardiac function and attenuated fibrosis while improving vascularization and cardiomyocyte survival in the marginal zone (Wu et al., 2020). In addition, it has been shown that ISX-9 is a small molecule with prosurvival, antioxidant, and regenerative properties, and that induction of CPCs by ISX-9 is an attractive cell-based cardiac regeneration therapy, and that these EVs contain a unique set of bioactive miRNAs, among which miR-373 has anti-fibrotic substantial effects. These new discoveries have important implications for preventing post-myocardial infarction remodeling (Xuan et al., 2019).

**FIGURE 2:**
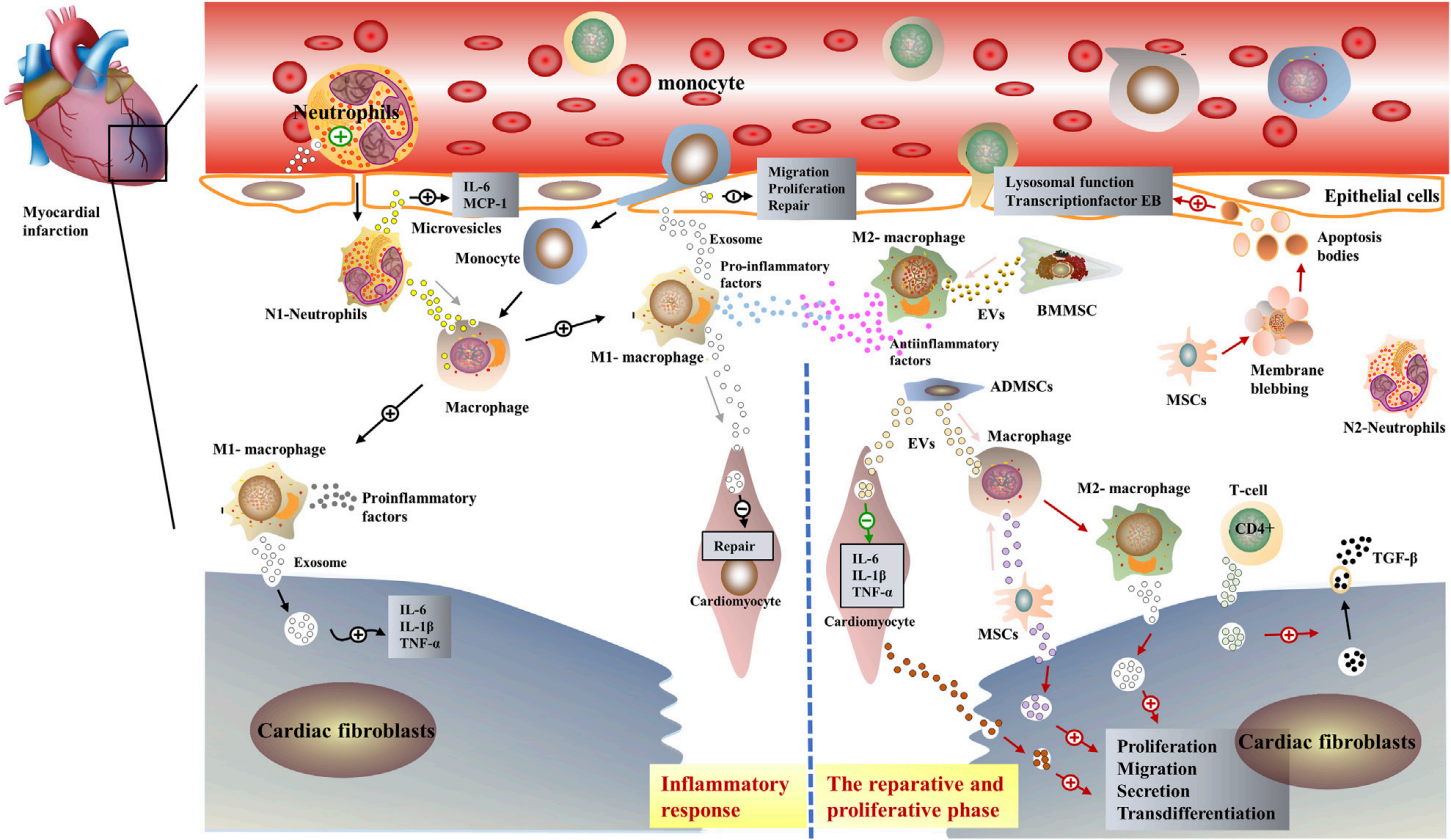
Schematic representation of the role of extracellular vesicles in post-infarction cardiac remodeling. EVs have similar biological properties to parental cells, in the early inflammatory phase post-infarction, immune cells such as pro-inflammatory M1-MØ and neutrophils, secret exosomes containing pro-inflammatory miRNAs. Interaction with myocardial fibroblasts and endothelial cells exacerbates the inflammatory response in the infarcted area (Xiong et al., 2021). Subsequently, anti-inflammatory M2-MØ and immune cell-derived exosomes such as neutrophils dominate, interacting with mesenchymal stem cells as well as cardiomyocytes to exert anti-inflammatory effects (Wu et al., 2019).

In preclinical studies, EVs derived from Cardiosphere-derived cells (CDCs) facilitated myogenesis and angiogenesis, ameliorated fibrosis, modulated immune responses, and generally improved cardiac function (Marban, 2018). MSCs mediate their disease-modifying biological activity by secreting paracrine factors, including EVs. Furthermore, like CDC-EVs, MSC-EVs contain a plethora of RNA species, like miRNAs. For instance, MSC-EVs from bone marrow-derived MSCs are enriched in miR-22 and have anti-fibrotic and anti-apoptotic properties in a mouse model of AMI (Feng et al., 2014). This demonstrates a significate role in regulating fibrotic repair after infarction by intervening in extracellular vesicle-loading cargos (Figure 2).

## 6 Therapeutic strategies for targeting extracellular vesicles in regulating cardiac remodeling after myocardial infarction

### 6.1 Engineered extracellular vesicle-based therapeutics

Although native EVs make great achievements in preclinical research, realizing the precisely controllable release of these cargos for the target regions remains a challenge (Fan Z et al., 2022). Accordingly, to enhance the ability of EVs to load cargoes, improve the controllability and precision of vesicular cargoes released in the infarct region, further attenuate inflammation and maintain the homeostasis of fibrotic scar repair following MI, researchers distill experiences and inspirations from nano materials-based drug delivery research and engineer modification of extracellular vesicles, including bioengineering, chemical engineering, and physical engineering (Man et al., 2020; Nasiri Kenari et al., 2020; Wu et al., 2021). Targeted manipulation of the extracellular vesicles’ genes and proteins is an essential part of bioengineering, which can direct EVs to a specific tissue or organ and increase their utilization. For instance, EVs derived from cardiosphere-derived stem cells are labeled by infarcted heart targeting peptide, so-called “cardiac-homing peptide”, the vesicles recruit to the infarct area and improve cardiac function by attenuating cardiac fibrosis, inducing angiogenesis, and promoting cardiomyocyte proliferation (Vandergriff et al., 2018). Meanwhile, there are also several studies demonstrated that HEK 293 cells transfecting with vectors encoding CTP-Lamp2b can derive exosomes with cardiac-targeting peptide (CTP)-Lamp2b on their membrane (CTP-Exo) (Kim et al., 2018), these vesicles can transport curcumin directly to the heart and improve cardiac function by regulating the PTEN/Akt/Bax signaling pathway associated with cardiomyocyte apoptosis *via* upregulating miR-144-3p (Kang et al., 2021). Furthermore, MSCs-derived exosomes modified with ischemic myocardium-targeting peptide CSTSMLKAC (IMTP) can specifically target ischemic myocardium, attenuating inflammation and apoptosis, promoting angiogenesis, and restoring cardiac function (Wang et al., 2018). On the other hand, Tian et al. found that exosomes (Hypo-Exo) derived from bone marrow mesenchymal stem cells (BMMSCs) under hypoxic conditions have excellent protections against ischemic diseases. The combination of Hypo-Exo with “CSTSMLKAC” peptide, an ischemic myocardial targeting peptide, significantly reduced apoptosis after MI *via* targeting Exo in ischemic heart regions, suggesting that IMT-Exo could be a novel drug carrier to enhance the specificity of drug delivery in ischemic diseases (Zhu et al., 2018). These findings demonstrated that the delivery of various cargoes in cardiac-targeted peptide-modified exosomes may be a promising strategy for MI treatment.

Alginate hydrogel possesses favorable biocompatibility and has been widely applied in tissue engineering and cellular engineering (Ruvinov and Cohen, 2016). Researchers have constructed an injectable conductive hydrogel to bind exosomes derived from human umbilical cord mesenchymal stem cells and injecting the hydrogel into injured rat hearts effectively prolongs the retention time of exosomes in the ischemic myocardium (Zou et al., 2021). Dendritic cell-derived exosomes (DEX) can activate Treg cells (Sakaguchi, 2004), and contribute to an early shift of macrophage subsets from an inflammatory M1 phenotype towards a reparative M2 phenotype after MI, thereby improving the immune microenvironment in the infarct area and offering the possibility of improving post-infarcted cardiac function. However, they have a short retention time and the therapeutic effects are transient. Accordingly, Researchers have developed a new drug delivery system that contains a sodium alginate hydrogel that continuously releases DEX, an approach that significantly increases therapeutic efficacy (Zhang et al., 2021). In addition, some investigators have also found that by combining the advantages of natural EVs and novel nanomaterials, engineered vesicles with biofilm/synthetic material chimeras were successfully constructed to enhance further the targeting and loading of natural vesicles with therapeutic molecules. Jin et al. found that engineered vesicles can effectively load microRNA-21 or curcumin for targeted delivery to achieve precise release of therapeutic molecules in macrophages, modulate the phenotypic transformation of macrophages, and effectively control inflammation levels (Andaloussi et al., 2013) (Table 1).

**TABLE 1:** Specific application and mechanism of miRNAs carried by EVs in post-infarction cardiac remodeling.

Stages	Species	Origin	Experimental model	Mechanism	References
Pro-inflammation	miR-155	M1 MØ - Exos	C57BL/6 J male mice	TNF-α↑	Wang et al., 2017; Liu S et al. (2020)
			MI model	IL-1β↑	
				CCL2↑	
	miR-328-3p	CMs-Exos	BALB/C nude mice	Activate Caspase facilitate apoptosis	Huang P et al. (2021)
			MI model		
	miR-146a	ADSCs-Exos	SD male rat	EGR1↓	Pan W et al. (2019)
			MI model		
	miR-375	MØ -Exos	C57BL/6J male mice	Stimulate M1 MØ polarization	Garikipati et al. (2017)
			MI model		
Anti-inflammatory	miR-25-3p	MSC-Exos	BALB/c male mice	Disinhibit the expression of SOCS3	Peng et al. (2020)
			I/R injury model		
	miR-126	ADSC- Exos	SD male rat	IL-1β↓	Luo et al. (2017)
			MI model	IL-6↓	
				TNF-α↓	
	miR-19a	hUCMSC-Exos	SD male rat	IL-1β↓	Huang P et al. (2020)
			AMI model	IL-18↓	
				JNK3/caspase-3↓	
	miR-181a	MSC-Exos	C57BL/6 male mice	Increase Tregs polarization	Wei et al. (2019)
			I/R injury		
	miR-181b	CDCs-Exos	WKY female rat	NF-κB ↓	de Couto et al. (2017)
			I/R injury	Polarize MØ to an M2 phenotype	
	miR-23a-3p	hUCMSC-Exos	C57BL/6J male mice	Inhibit ferroptosis	Song et al. (2021)
			AMI		
	miR-22	MSC-Exos	C57BL/6J male mice	Reduce apoptosis	Feng et al. (2014)
			MI model		
	miR-182	MSC-Exos	C57BL/6 mice	Polarize MØ to an M2 phenotype	Zhao et al. (2019b)
			I/R injury		
	miR-93-5p	ADSC-Exos	SD male rat	TLR4/NF-kB ↓	Liu J et al. (2018)
			AMI model		
	miR-129-5p	BMSCs-Exos	C57BL/6J male mice	Inflammatory cytokines↓	Wang et al. (2022)
			MI model		
	miR-24-3p	UMSC-Exos	—	NF-kB ↓	Zhu et al. (2022)
				Polarize MØ to an M2 phenotype	
	miR-200b-3p	MSCs-EVs	C57BL/6J male mice	BCL2L11↓	Wan et al. (2022)
			MI model		
	miR-223	hUCMSCs-EVs	SD male rat	TNF-α↓	Yang et al. (2022)
			MI model	IL-6↓	
				IL- 1β↓	
	miR-1271-5p	M2 MØ -Exos	C57BL/6J male mice	SOX6↓	Long et al. (2021)
			AMI model		
	miR-302d-3p	MSC-EVs	C57BL/6J male mice	TNF-α↓	Liu et al. (2022)
			AMI model	IL-6↓	
**Pro-fibrosis**	miR-142-3p	CD4+T cell	C57BL/6J male mice MI model	Activate myofibroblast	Cai et al. (2020)
		Exosome			
	miR-218-5p	EPC-Exos	SD rat	Promote CFs proliferation	Ke et al. (2021)
			MI model		
	miR-363-3p	EPC-Exos	SD rat	Promote CFs proliferation	Ke et al. (2021)
			MI model		
	miR-494-3p	DC-Exos	C57BL/6J male mice MI model	Promote angiogenesis	Liu Y et al. (2021)
	miR-146a	ADSC-Exos	SD male rat	EGR1↓	Pan J et al. (2019)
			MI model		
	miR-92a	CMs-Exos	C57BL/6J male mice MI model	α-SMA↑ periostin↑	Wang X et al. (2020)
	miR-142-3p	CD4^+^ T-cellT-cell	C57BL/6J male mice MI model	β-catenin↑	Cai et al. (2020)
		Exosome		Col1a1↑	
				Col3a1↑	
				α-SMA↑	
**Anti-fibrosis**	miR-155	M1 MØ - Exos	C57BL/6J male mice MI model	Inhibit the proliferation of CFs	Wang et al. (2017)
	miR-126	ADSC-Exos	SD male rat	Inhibit the tissue fibrosis	Luo et al. (2017)
			MI model		
	miR-22	MSC-Exos	C57BL/6J male mice	Reduce fibrotic area	Feng et al. (2014)
			MI model		
	miR-1246	EPC-Exos	SD male rat	Change of Fibroblasts to Endothelial Cells	Huang Y et al. (2021)
			MI model		
	miR-1290	EPC-Exos	SD male rat	Changes of Fibroblasts to Endothelial Cells	Huang J et al. (2021)
			MI model		
	miR-19a/19b	BMMSC-Exos	SD male rat	Reduce fibrotic area	Wang S et al. (2020)
			MI model		
	miR-29b-3p	BMSCs-Exos	SD male rat	Inhibit the proliferation, migration, and differentiation of CFs	Zheng et al. (2022)
			MI model		
	miR-106a–363	iCMs-EVs	SCID mice	Repressing Notch3	Jung et al. (2021)
			MI model		
	miR-4732-3p	MSC-EVs	Nude rats	Inhibit MFBs differentiation and the production of extracellular matrix	Sanchez-Sanchez et al. (2021)
			MI model		
	miR-212-5p	MSC- EVs	C57BL/6J male mice	COl I↓	Wu et al. (2022)
			MI model	α-SMA↓	
	miR-223	hUCMSCs-EVs	SD male rat	COl I↓	Yang et al. (2022)
			MI model	COl III↓	
	miR-302d-3p	MSC- EVs	C57BL/6J male mice	Inhibit MFBs differentiation	Liu et al. (2022)
			AMI model		

### 6.2 Drug pretreatment of extracellular vesicles to enhance its therapeutic effect

In addition to the engineered modification of EVs, several types of research focus on the drug regulation of EVs and make several significant findings.

Numerous experimental and clinical studies demonstrate that myocardium is protected from ischemic left ventricular remodeling by long-term antagonisty of the purinergic GPCR P2Y12 (Roubille et al., 2012; Nanhwan et al., 2014; Vilahur et al., 2016; Bansilal et al., 2018). Ticagrelor is a selective and reversible P2Y12 receptor antagonist that induces exosome release from CPCs, resulting in the release of anti-apoptotic HSP70. Simultaneous continuous pretreatment of cardiomyocytes with HCPC derived exosomes exposed to low-dose of ticagrelor attenuates hypoxia-induced apoptosis *via* acute phosphorylation and activation of ERK42/44, resulting in myocardial protection effect (Casieri et al., 2020). This research has clinical implications for enhancing the endogenous exosomal anti-hypoxic response and prevent the heart from ischemic injury by developing new non-invasive pharmacological approaches. In addition, atorvastatin pretreatment has been demonstrated to enhance the function of BMMSC-derived exosomes in angiogenesis, cardiomyocyte protection, and long non-coding RNA H19 (lncRNA H19) is a mediator of regulating miR-675 expression and promoting atorvastatin pretreatment MSC-Exo (MSC^ATV^-Exo) effect in angiogenesis. Importantly, lncRNA H19 and its downstream signaling pathways mediate the cardioprotective effects of MSC^ATV^-Exo (Huang L et al., 2020). Other studies have shown that sulpiride/valsartan can improve cardiac function and ameliorate myocardial fibrosis by downregulating miR-181a in exosomes in a rodent chronic MI model (Vaskova et al., 2020).

TCM has been practiced and developed for thousands of years. In recent decades, considerable progress and achievements have been made in studying the mechanism of TCM using modern molecular biology techniques (Zhou et al., 2016). One of the hotspots is the research on the targeting and regulation of EVs using TCM, such as the regulation of stem cells and immune cells. Stem cell transplantation is a promising therapeutic alternative to facilitate myocardial repair following MI, however, its clinical application is limited by the low preservation and survival rates of implanted cells (Feyen et al., 2016). In recent years, new strategies have been developed, including combined cell therapy with BMMSC exosomes, which have been demonstrated that such strategies have anti-apoptotic, anti-inflammatory, and pro-angiogenic effects. Transplantation of exogenous vesicles into the ischemic heart within 30 min of MI significantly modulates the ischemic environment, including decreasing inflammatory IL-6 and TNF-α, enhancing SDF-1 expression and MSC survival. Additionally, several studies have demonstrated that combined pretreatment with hypoxia and the herbal compound Tongxinluo can effectively achieve better performance in facilitating cardiac repair through increasing CXCR4 expression, which provides new insights into stem cell therapy for cardiac rehabilitation (Xiong et al., 2022). Furthermore, Ruan et al. suggested that Suxiao Jiuxin pills modulate myocardial MSC-Exos, causing structural genetic chromatin remodeling in recipient cardiomyocytes and accelerating cardiomyocyte propagation (Ruan et al., 2018). Accordingly, researchers rolled out several studies to enhance EVs targeting, thus making them better delivery vehicles (Veerman et al., 2019).

## 7 Clinical applications and regulation of extracellular vesicles

Clinical applications of EVs are novel therapeutic modalities, including vaccines, diagnostic criteria, and drug delivery. The idea of using EVs as anti-tumor vaccines originated in the last century. Basic studies suggested that DEX can promote T-cell dependent antitumor effect, and phase I clinical trials (to vaccinate peptide-pulses DEX for patients with cancer) demonstrated that the feasibility and safety of inoculation of DEX (Escudier et al., 2005; Morse et al., 2005). Immediately after, Sophie et al. developed a second-generation DEX with enhanced immunostimulatory properties and produced large-scale IFN-γ-DEX vaccines while conducting phase II clinical trials (NCT01159288) under the guidance of Institut Gustave Roussy in France, in which DEX was administered to patients with non-small cell lung cancer with the aim of increasing progression-free survival (Viaud et al., 2011; Lener et al., 2015). In respiratory diseases, preclinical studies have established that MSC-Exos can ameliorate most of the pathological changes caused by lung infections. And a clinical study (NCT04602104) for treating acute respiratory distress syndrome (ARDS) with MSC-Exos Nebulizer is currently recruiting patients, this study explores a new approach to treat ARDS and assesses its safety by administering human mesenchymal stem cell exosomes (hMSC-Exos) aerosol inhalation to patients. It has also been shown that ExoFlo™, derived from allogeneic bone marrow mesenchymal stem cells, can reconstitute immunity, downregulate cytokine storm and restore oxygenation in patients with severe COVID-19, demonstrating the promise of ExoFlo for the treatment of COVID-19 (Sengupta et al., 2020). In renal disease, intra-arterial and intravenous administration of MSC-Exos to patients with grade III-IV chronic kidney disease in phase II/III clinical trials was shown to ameliorate inflammation and renal function. No adverse events associated with MSC-Exos administration were observed in subjects during the 1-year follow-up period (Nassar et al., 2016; Guo et al., 2020). In cardiovascular system diseases, a phase II randomized clinical trial of intravenous ischemia-tolerant MSCs (itMSCs) in patients with non-ischemic cardiomyopathy showed that single-dose of intravenous itMSCs was safe and well tolerated compared with controls, increasing the patient’s 6-min walk distance while eliciting systemic immunomodulatory effects associated with improved LVEF (Butler et al., 2017). Examples of these clinical trials showed the great therapeutic potential of EVs.

**FIGURE 3:**
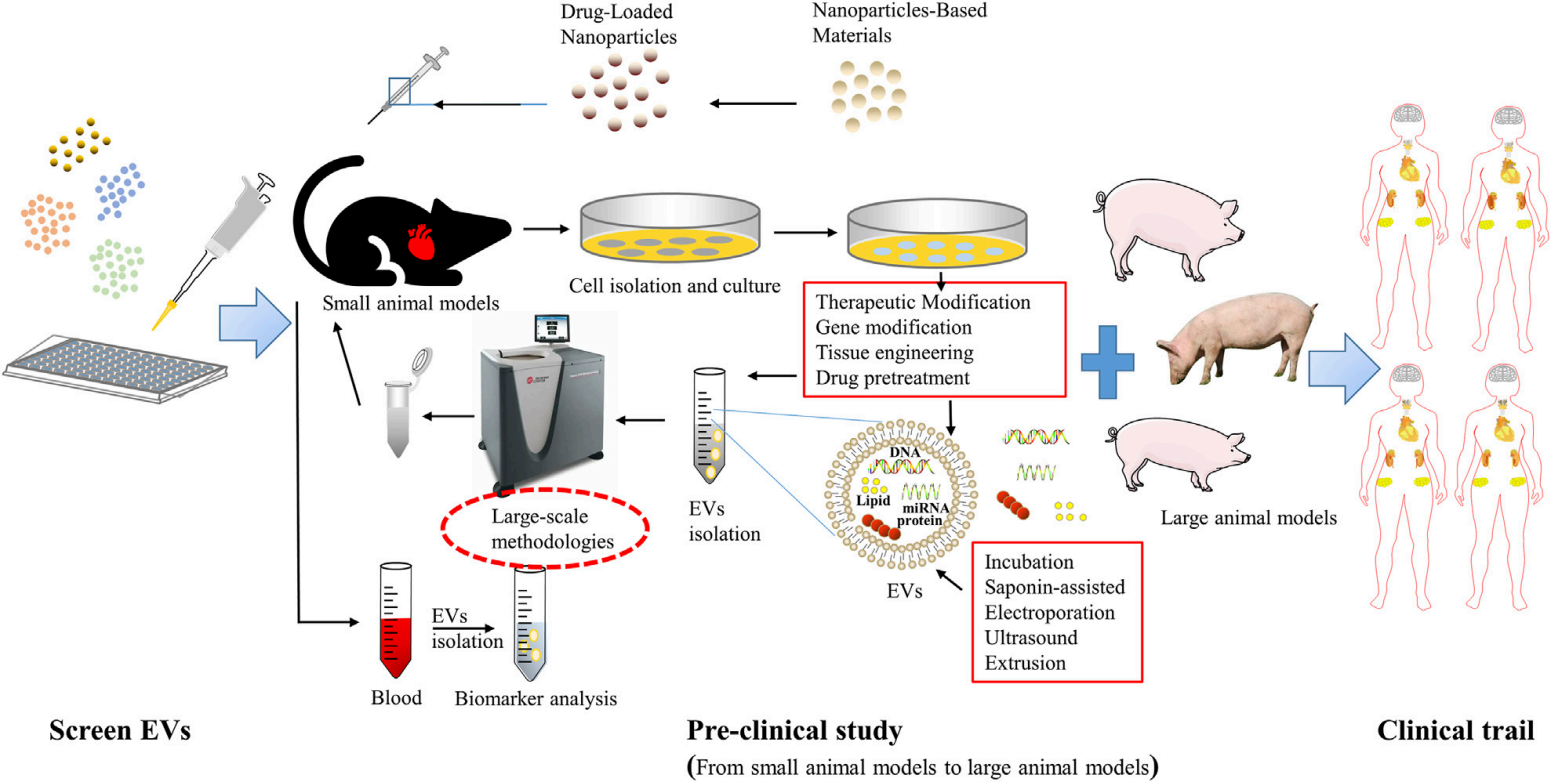
Extracellular vesicle-based therapeutics toward clinical application. The first is a basic study to identify potential EVs, followed by preclinical studies, including small animal *in vivo* trials and large animal *in vivo* trials, as well as researches to evaluate the manufacturing process, quality control, and stability (CMC); and finally a phase III randomized clinical trial to further evaluate its safety and efficacy.

The complex properties of EVs make their specification and characterization difficult. Thus, in addition to controlling the final product by specification and characterization, the quality of EVs must be ensured by quality control of the raw material and regulation of the manufacturing process (Tsuchiya et al., 2022). EV manufacturing must be performed in accordance with Good Practice (GxP) regulations, which are a collection of quality guidelines and regulations designed to ensure that biological/medical products are safe, meet their intended use and follow quality processes in manufacturing, control, storage and distribution (Good Manufacturing, Good Laboratory, Good Distribution, Good Clinical, Good Scientific Practice) (Soekmadji et al., 2020). The biological activity of EV used as a therapeutic agent must be tested in a qualified bioassay, called a “potency assay" (Lener et al., 2015). EVs are subject to detailed guidelines for the clinical application and manufacture of novel biomedical products in the EU. In Australia, the government’s Therapeutic Goods Administration (TGA) office provides rules and guidelines related to the manufacture and use of therapeutic agents often adopted in EU rules. In the United States, EV-based therapeutics for human use are regulated by the Center for Biologics Evaluation and Research (CBER) within the Food and Drug Administration (FDA) (Lener et al., 2015). (Figure 3).

## 8 Conclusion

Post-infarction cardiac remodeling is a complex and diverse pathological process consisting of two main phases: inflammatory response and fibrotic scar repair. Sudden and substantial cardiomyocyte death triggers a vigorous inflammatory response and subsequently activated MFBs, which secrete collagen, leading to myocardial fibrosis and consequent scar formation. Moderate myocardial fibrosis plays a critical protective role, maintaining the structural integrity of the chambers and preventing cardiac rupture, while persistent myocardial fibrosis can lead to cardiac stiffness and diastolic dysfunction, eventually causing HF. The search for new measures and approaches that can attenuate the post-infarction inflammatory response as well as maintain the dynamic balance of fibrosis repair is particularly critical. EVs of different cellular origins play a dual role in the early post-infarction inflammatory response and the subsequent repair of fibrotic scars. During the early inflammatory phase, these vesicles are involved in both pro-inflammatory and anti-inflammatory responses, and therefore, by achieving effective control of the release of pro-inflammatory vesicles and the delivery of their cargoes is of great importance to contain the early adverse persistent inflammatory response after infarction. At the same time, these vesicles play a dual role of pro-fibrotic and anti-fibrotic effects in the subsequent fibrotic scar repair phase, a property that also shows the importance of maintaining the dynamic balance of post-infarction fibrotic repair by modulating EVs. Currently, research on targeted regulation of EVs in cardiovascular diseases has focused on drug pretreatment as well as engineered modifications.

Research associated with the intercellular delivery of functional nucleic acids and proteins by EVs has demonstrated their advantages as cargo carriers, such as low immunogenicity, high stability and biocompatibility, long cycle life, and ability to cross the blood-brain barrier. These properties make EVs an important endogenous carrier for delivering therapeutic drugs, however, the current research on extracellular vesicle drug delivery is mainly focused on cancer and neurological related diseases, while preclinical studies on cardiovascular diseases are rare. The reasons for this are limited by the isolation of endogenous vesicles, storage, mass production techniques, low efficiency of targeted drug delivery and cellular uptake, on the one hand, and the unknown biological distribution of drug-loaded vesicles in the individuals treated with them, on the other hand, which may lead to difficult challenges in managing the use of exosomes in standardized methods related to their isolation, quantification and outcome analysis. Currently, EVs were isolated by Polymer precipitation, Differential ultracentrifugation, Field-flow fractionation, and Gradient density ultracentrifugation. These methods always lacking in yield and purity. Distinct purification methods of EVs exist in relation to the physical or molecular characteristics of isolated EVs preparations and affect the results of downstream analysis. The best separation strategy for EVs is by applying different methods of isolation in order to achieve a desirable recovery and purity and report in detail about standardized approaches for isolation, this research is particularly important, especially in the context of promising clinical applications of EVs(Clos-Sansalvador et al., 2022). Second, drug loading cannot disrupt the membrane structure and content of EVs, and finding effective ways to load therapeutic drugs into these EVs remains a great challenge. Although, the use of EVs as drug delivery carriers is still in the developmental stage. However, with a deeper understanding of the physiological properties of EVs, improved isolation and drug delivery techniques for EVs, the understanding and conclusion of exosome-loaded drugs in other diseases (cancer, hepatitis, Parkinson’s disease, *etc.*) will help develop or expand future therapeutic approaches for post-infarction cardiac remodeling, mitigate the inflammatory response during post-infarction cardiac remodeling, precisely regulate the dynamic balance between pro- and anti-fibrosis, and ultimately the transition from basic research to clinical applications.

## Glossary

AMI: acute myocardial infarction
EVs: Extracellular vesicles
HF: heart failure
MI: myocardial infarction
I/R: ischemia-reperfusion
DAMPs: damage-associated molecular patterns
CFs: cardiac fibroblasts
MSCs: mesenchymal stem cells
CPCs: cardiac progenitor cells
MVs: microvesicles
ABs: apoptotic bodies
RIPK3: receptor-interacting protein kinase three
RIPK1: receptor-interacting protein kinase 1
PRRs: pattern recognition receptors
TNF: tumor necrosis factor
IL-1β: interleukin 1β
IL-6: interleukin 6
MCP-1: monocyte chemotactic protein-1
ATP: adenosine triphosphate
NF-κB: nuclear factor κB
ECs: endothelial cells
PSCs: progenitor stem cells
MFBs: myofibroblasts
TGF-β: transforming growth factor β
ECM: extracellular matrix
ILVs: intraluminal vesicles
MVBs: multivesicular bodies
PS: phosphatidylserine
ESCRT: endosomal sorting complex required for transport
CAMs: cell adhesion molecules
CCL3: macrophage inflammatory protein 1 α
IL-1b: interleukin 1b
IL-12a: interleukin 12a
TNF-α: tumor necrosis factor
IL-10: interleukin 10
CCL-2: chemokine (C-C motif) ligand 2
NDMVs: neutrophil-derived MVs
NDTRs: neutrophil-derived trails
AnxA1: membrane-linked protein A1
LMVs: leukocyte-derived MVs
TGF-β1: transforming growth factor β1
DCs: dendritic cells
ox-LDL: oxidized low-density lipoprotein
BMMSC: Bone marrow mesenchymal stem cell
ADSCs: adipose stromal cells
Dyrk: bispecific tyrosine phosphorylation kinase
MSC-Exos: MSC-derived exosomes
hUCMSC-Exos: human umbilical cord mesenchymal stem cell-derived exosomes
EPCs: endothelial progenitor cells
CD31: cluster of differentiation 31
TCM: traditional chinese medicine
C-MSCs: cardiac mesenchymal stem cells
TFEB: transcription factor EB
CDIP1: cell death involved p53 target 1
α-SMA: α-smooth muscle actin
Col I: collagen type I
MSC-EVs: MSC-derived extracellular vesicles
PDGFR-β: platelet-derived growth factor receptor-β
HPSC: human pluripotent stem cell
CVPCs: cardiovascular progenitor cells
CHP: cardiac homing peptide
CDCs: cardiosphere-derived cells
PCs: progenitor cells
iCMs: human iPSC-derived cardiomyocytes
IMTP: ischemic myocardium‐targeting peptide
AMSC-Exos: adipose mesenchymal stem cell-derived exosomes
EGR1: growth response factor 1
M1-MØ: M1 macrophages
M2-MØ: M2 macrophages
ADSCs-Exos: exosomes secreted by adipose-derived stem cells
CDCs-Exos: exosomes secreted by Cardiosphere-derived cells
EPC-Exos: exosomes secreted by endothelial progenitor cells
BMMSC-Exos: exosomes derived from bone marrow mesenchymal stem cells
DC-Exos: dendritic cells derived exosomes
CMs-Exos: cardiomyocytes-derived exosomes
UMSC-Exos: umbilical cord MSC-derived exosomes
DEX: dendritic cell-derived exosomes
ARDS: acute respiratory distress syndrome
hMSC-Exos: human mesenchymal stem cell exosomes
itMSCs: ischemia-tolerant MSCs
